# Individuals with a ventral hernia who report moderate to high fear have worse functional performance than those with low fear

**DOI:** 10.1007/s10029-024-02979-0

**Published:** 2024-02-26

**Authors:** Elanna K. Arhos, Benjamin K. Poulose, Stephanie Di Stasi, Ajit M. W. Chaudhari

**Affiliations:** 1 School of Health and Rehabilitation Sciences, Ohio State University, 2835 Fred Taylor Drive, Columbus, OH 43202, USA; 2 Ohio State University Wexner Medical Center, Sports Medicine Research Institute, Columbus, OH, USA; 3 Department of Surgery, Division of General and Gastrointestinal Surgery, Center for Abdominal Core Health, Ohio State University Wexner Medical Center, Columbus, OH, USA; 4 Division of Physical Therapy, School of Health and Rehabilitation Sciences, Ohio State University, Columbus, OH, USA

**Keywords:** Hernia, Physical therapy, Rehabilitation, Kinesiophobia

## Abstract

**Purpose:**

Ventral hernia repairs (VHR) are performed to restore the integrity of the abdominal wall. Fear of movement, or kinesiophobia, may develop in patients with ventral hernia due to pain and functional impairments, however it has not yet been objectively measured in this patient population. The purpose of this study was to test the hypothesis that in patients with ventral hernia awaiting surgical repair, higher levels of kinesiophobia would be associated with poorer mobility, abdominal core function, and quality of life.

**Methods:**

Seventy-seven participants scheduled for ventral hernia repair were enrolled as part of an ongoing randomized controlled trial (NCT05142618). The Tampa Scale of Kinesiophobia (TSK-11) is an 11-item questionnaire that asks about fear of movement and physical activity restriction. Participants were split into groups based on their TSK-11 score (minimal, low, moderate to high). Primary outcome measures included the five-time sit-to-stand (5xSTS), Quiet Unstable Sitting Test (QUeST), and the Hernia-Related Quality-of-Life (HerQLeS) survey. A one-way ANOVA with a Bonferroni correction compared QUeST, 5xSTS, and HerQLes results between groups.

**Results:**

Groups were significantly different on 5xSTS (minimal: 11.4 ± 2.6 s, low: 13.8 ± 3.1 s, moderate to high: 17.8 ± 9.8 s; *p* = 0.001) and HerQLes (minimal: 58.0 ± 27.8, low: 49.4 ± 22.0, moderate to high: 30.6 ± 25.3; *p* = 0.003) but not QUeST (minimal: − 2.8 ± 2.5, low: − 6.8 ± 10.0, moderate to high: − 5.5 ± 5.0; *p* = 0.16).

**Conclusion:**

Individuals with moderate to high kinesiophobia have worse pre-operative performance-based (5xSTS) and self-reported (HerQLes) function and quality of life than those with minimal and low kinesiophobia. Future research should examine the influence of kinesiophobia on post-operative outcomes as it may be a potent target for rehabilitation.

## Introduction

Ventral hernia repairs (VHR) are one of the most common surgeries performed in the United States, with up to 600,000 repairs occurring yearly [1]. Prior to surgery, a majority of patients will experience pain from their hernia [2], which can have a significant impact on daily function and quality of life [3]. Fear of movement, or kinesiophobia, may develop in patients with ventral hernia and lead to changes in behavior such as avoiding certain movements and reducing physical activity. In individuals with ventral hernia, pre-operative exercise level is associated with lower rates of post-operative complications, suggesting pre-operative physical activity may be an important target for improving post-operative outcomes [4]. Over time, fear-avoidance behavior due to pain may contribute to continued functional impairments and negatively affect recovery.

Kinesiophobia is defined as “an excessive, irrational, and debilitating fear of physical movement and activity resulting from fear of painful injury or re-injury.”[5] High levels of kinesiophobia have been shown to impact function [6–8], pain [9], and self-reported outcomes[10] in a variety of patient populations. Kinesiophobia has also been linked to changes in movement strategies, primarily manifesting as restricted movement and muscle activation patterns [11]. The Tampa Scale of Kinesiophobia uses a questionnaire to assess pain-related fear of movement, with lower scores suggesting less fear [12, 13]. Understanding the contribution of kinesiophobia to the clinical presentation of patients with ventral hernia will assist clinicians in developing pre- and post-operative rehabilitation interventions targeting pain-related fear of movement. Similarly, characterizing pre-operative kinesiophobia may be important for clinicians treating patients with ventral hernia and inform patient education around fear of movement prior to proceeding with surgical treatment.

Patients with ventral hernia report reduced function and quality of life [3, 14, 15], however there are few performance-based tests of physical function assessed in a clinical setting. The associations between pre-operative kinesiophobia and functional measures remains unknown in this clinical population. Therefore, the purpose of this study was to compare function and quality of life in patients with ventral hernia between different categories of kinesiophobia. We tested the hypothesis that these measures would be worse in patients who report higher levels of kinesiophobia.

## Methods

Participants were involved as part of an ongoing randomized controlled trial (NCT05142618) designed to test the initial feasibility and efficacy of post-operative physical therapy (PT) [16]. All participants provided informed consent, and The Ohio State University Institutional Review Board approved this study. The funders played no role in the design, conduct, or reporting of this study. Participants must have been between 18–70, diagnosed with a ventral hernia, and scheduled for elective VHR. Participants were excluded if they had a transverse hernia width < 2 cm, have a previously diagnosed movement or balance disorder, use an ambulatory assistive device, or were currently participating in PT or other skilled exercise intervention supervised by a medical rehabilitation professional at the time of the eligibility assessment. Participants included in this analysis were at the pre-operative timepoint and had not yet received surgery or randomization to one of the treatment arms.

Primary outcomes for this secondary study included the Five Time Sit to Stand (5xSTS), Quiet Unstable Sitting Test (QUeST) and the Hernia-Related Quality-of-Life (HerQLes). The 5xSTS was assessed as the time (nearest 0.1 s) taken to stand up and sit down from a standard height chair five times. It is a reliable measure with previously established normative values [17–20]. The minimal clinically important difference (MCID) of the 5xSTS is 2.3 s.[20] The QUeST is an assessment of abdominal core function using postural sway while seated on an unstable surface, eyes closed, with a cognitive dual task [21]. The QUeST assesses mean center of pressure path length during sitting balance using a force plate under a BOSU^®^ over three averaged trials. The QUeST core stability score reported is the absolute difference from the normative population mean in multiples of the 95% confidence interval of the standard error of measurement, where 0 represents normal and < 0 represents lower core stability [21]. Finally, the HerQLes is a 12-item survey of quality of life for patients with hernia disease scored from 0–100, where higher scores indicate higher quality of life [22].

Participants were split into three groups based on their Tampa Scale of Kinesiophobia (TSK-11) score, an 11-item self-reported questionnaire score out of 44 possible points asking about fear of movement and physical activity participation restrictions due to pain. Groups were split according to previous literature as minimal (TSK ≤ 22), low (TSK 23–28), or moderate to high kinesiophobia (TSK ≥ 29) [23].

All statistical analyses were performed using SPSS (IBM Corporation, Chicago, IL). All variables of interest were assessed for normality using Shapiro–Wilk analysis. Demographic characteristics were compared between the minimal, low, and moderate to high kinesiophobia groups using Chi square test of proportions and one-way analysis of variance (ANOVA) models with parametric data or Kruskal–Wallis models when data was not parametric. A Kruskal–Wallis test was run to compare QUeST, 5xSTS, and HerQLes results between groups. Significant between group differences were followed up with Dunn post-hoc analysis. Alpha level was set to *p* < 0.05 a priori.

## Results

**Fig. 1:**
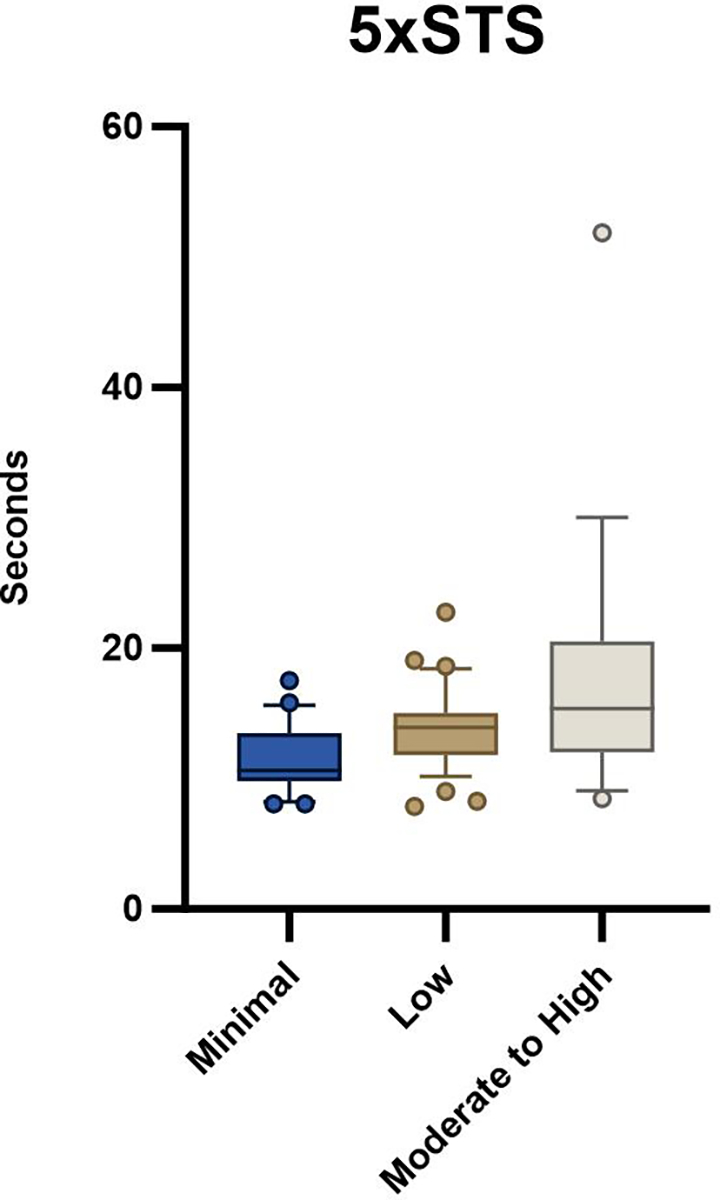
5xSTS outcome between minimal, low, and moderate to high kinesiophobia groups; whiskers indicate the 10th to the 90th percentile

**Fig. 2:**
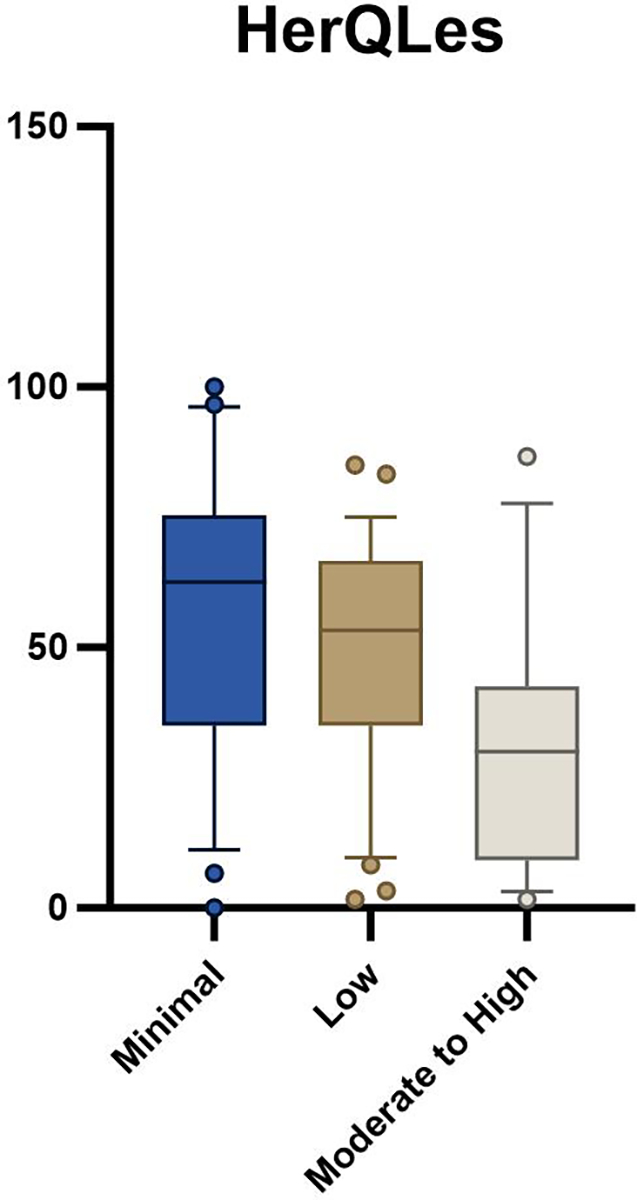
HerQLes outcome between minimal, low, and moderate to high kinesiophobia groups; whiskers indicate the 10th to the 90th percentile

**Fig. 3:**
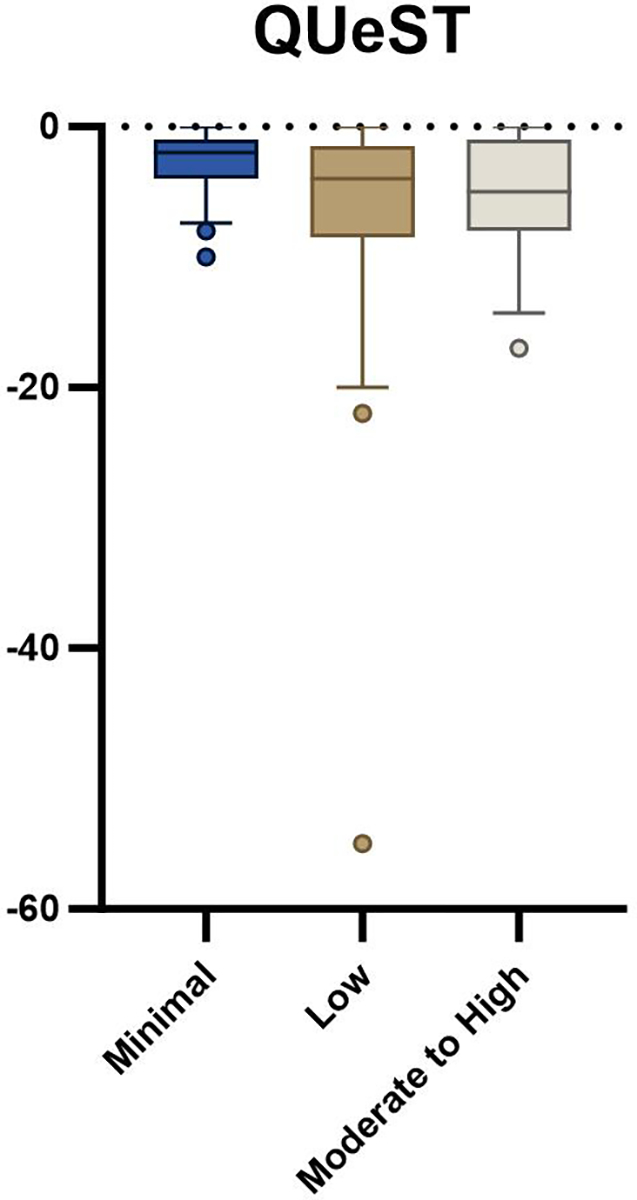
QUeST outcome between minimal, low, and moderate to high kinesiophobia groups; whiskers indicate the 10th to the 90th percentile

Seventy-seven participants awaiting ventral hernia repair were enrolled in the parent trial at the time of this analysis. Baseline demographic variables were not different between groups (Table 1). There was a statistically significant difference between groups with the 5xSTS (*p* = 0.001, Table 2, Fig. 1) and HerQLes (*p* = 0.003, Table 2, Fig. 2) but not the QUeST (*p* = 0.24, Table 2, Fig. 3). Post-hoc comparisons revealed that the moderate to high fear group performed significantly worse on the 5xSTS compared to both the minimal (*p* = < 0.001) and low fear group (*p* = 0.007) and significantly worse on the HerQLes compared to both the minimal (*p* = 0.002) and low fear (*p* = 0.018) groups.

## Discussion

We found that individuals with moderate to high kinesiophobia demonstrated significantly worse pre-operative function and quality of life than those in the minimal and low kinesiophobia groups. The findings from this study supported our hypothesis that patients with higher levels of pre-operative kinesiophobia have worse functional performance and self-reported quality of life than patients with minimal to low kinesiophobia. These results suggest that kinesiophobia is an important patient-related factor in individuals with ventral hernia that may help to distinguish those with poor physical function and quality of life.

Assessing kinesiophobia is of relevance as patients may develop fear-avoidant behavior, which ultimately can lead to reduced physical activity and changes in movement [11, 23]. Reducing physical activity after being medically diagnosed with a hernia is common, with a large nationwide survey suggesting 32% of post-operative patients with parastomal hernias reporting that they are ‘much less active’ than prior to their surgery [24]. These data are concerning as low levels of physical activity put individuals at a greater risk for comorbidities and chronic health conditions (e.g., diabetes, stroke). In the same survey, 69% of patients did not realize engaging in abdominal or core exercises was important, which suggests a gap between patient education and physical activity in this patient population [24]. The persistence of fear-avoidant behavior post-operatively could be detrimental to recovery, causing patients to become less physically active and reduce their adherence to post-operative rehabilitation protocols.

While kinesiophobia may be an expected response after a medical diagnosis or surgical procedure, it can become problematic when it impacts function. In patients with Achilles tendinopathy, patients with higher levels of kinesiophobia had less calf muscle endurance recovery after exercise therapy compared to patients with lower kinesiophobia [25]. The lower levels of function in the group with moderate to high kinesiophobia in our cohort is problematic. The 5xSTS is a measure of functional lower extremity strength, activities of daily living, and balance and a mean of 19 s to complete the 5xSTS for patients in their 50 s and 60 s far exceeds the age-matched normative scores of 11.4 s.[26] This test was intended to assess fall risk in older adults and patients with balance disorders [27], however it has also been used to assess function and response to rehabilitation in a variety of clinical populations [20, 28]. The mean 5xSTS time of 13.4 s in the low kinesiophobia group also exceeded the normative values, making the minimal kinesiophobia group the only group to be within age-matched normative values of the 5xSTS [26]. Further, group differences between minimal and moderate to high kinesiophobia groups exceeded the 5xSTS MCID of 2.3 s [20]. These data indicate that even low levels of fear show functional deficits in adults with ventral hernia.

Age-matched normative values for the HerQLes do not exist, though it is worth noting that the moderate to high kinesiophobia group had significantly lower scores than both the low and minimal kinesiophobia groups, both of which surpassed the established MCID of 15.6. This MCID is based on a significant change in the HerQLes from baseline (pre-operatively) to 1-year post-operatively [29], and suggests a clinically meaningful difference between the patient’s perceived quality of life in the moderate to high kinesiophobia group compared to both the low and minimal kinesiophobia groups. Similarly, to the HerQLes, there are no age-matched normative values for the QUeST, however there was a similar trend as both the HerQLes and 5xSTS where the moderate to high kinesiophobia group had the worst outcomes compared to the minimal and low kinesiophobia groups. The QUeST is a feasible method of assessing abdominal core stability and can differentiate between patients with hernia and a control group with normal abdominal core health, where the median of a control group has been shown to be − 0.5, compared to a median score of − 2 in individuals with ventral hernia [21]. In the current study, the scores ranged from a mean of − 3.3 (low kinesiophobia group) to − 5.9 (moderate to high kinesiophobia group), suggesting a potential clinically meaningful difference in core stability. The QUeST and HerQLes scores were not related in the current study (r = − 0.83, *p* = 0.47), which has also been shown in previous literature [21], and this suggests that these tests assess different domains. Similarly, QUeST was not related to 5xSTS (r = − 0.75, *p* = 0.52), indicating that these outcome measures also assess different domains of function.

The moderate to high kinesiophobia group represented the patient sample with the worst performance-based function and self-reported quality of life and may be a group to specifically target with post-operative rehabilitation. The purpose of rehabilitation (i.e., PT) is to restore and optimize function by targeting individual post-operative impairments. It is currently unknown how rehabilitation will affect both kinesiophobia and function for individuals with ventral hernia, but there is evidence to suggest that PT intervention can affect psychologic measures in other clinical populations. In individuals with chronic low back pain, a clinical group that often presents with kinesiophobia [30], a multidisciplinary program including exercise and cognitive behavioral therapy was effective in reducing fear-avoidance beliefs and pain and the effects lasted a year after the intervention period ended [30]. A recent systematic review noted that multi-modal therapies were more effective than uni-modal therapies in reducing fear-avoidance beliefs and pain and improving quality of life in patients with musculoskeletal pain conditions [31]. These data suggest that while rehabilitation may support restoration of function, there may be value in future studies assessing a multi-modal (e.g., psychologically informed) rehabilitation approach for patients with ventral hernia.

Finally, increased kinesiophobia may be related to persistence of symptoms and could have a role in identifying individuals likely to experience a hernia recurrence. After anterior cruciate ligament reconstruction (ACLR), Baez et al. found that for every unit increase in injury related fear, participants had an 8.6 times greater odds of having persistent knee symptoms after surgery, and for every unit increase in kinesiophobia measured using the TSK-11, participants had a 3.9 times greater odds of persistent knee symptoms after surgery [32]. These data suggest there may be a relationship between fear-avoidance behavior and poorer recovery, which may relate to reduced participation within physical activity in order to avoid discomfort. In individuals with knee osteoarthritis, a chronic musculoskeletal disease process that commonly affects adults in the general population similar to those in our study, the presence of kinesiophobia measured using the TSK-11 was related to reduced quality of life and increased disability [33]. Similarly, in individuals following total knee replacement, higher TSK-11 scores were related to poor functional outcomes through 1-year post-operatively [34]. Continued research is warranted to unpack the relationship between kinesiophobia and functional performance in those with ventral hernia to ultimately determine how PT may be useful in optimizing patient recovery.

### Limitations

There are several limitations to consider when interpreting results from this study. First, while the four categories (minimal, low, moderate, high) of kinesiophobia have been validated in other clinical populations, they have not been validated within individuals with ventral hernia. There was no a priori power analysis as this was a secondary analysis of an RCT, so results should be interpreted with caution. Similarly, because few patients were in the high fear kinesiophobia category, we combined the moderate and high categories to keep sample sizes consistent across groups. While the QUeST has been found reliable in a developmental cohort of individuals with ventral hernia, it has not been validated in individuals with ventral hernia and does not yet have established MCIDs. Finally, there are currently no performance-based measures of function within this patient population to validate the QUeST against, and future work will use data from the parent ABVENTURE-P clinical trial to validate the QUeST and establish MCIDs.

## Conclusion

While TSK-11 has not previously been measured in this patient population, clinicians treating individuals with ventral hernia may consider assessing for kinesiophobia and its consequent relationship to physical function and compensatory movement strategies to direct effective rehabilitation.

## Figures and Tables

**Table 1.** Demographic variables between minimal, low, and moderate to high kinesiophobia groups. Results are mean ± standard deviation (SD).

	Minimal (n=22)	Low (n=37)	Moderate to High (n=18)	P-value

*Age*	48.9 ± 11.3	52.2 ± 9.5	47.5 ± 12.0	0.23
*Sex (F)*	8	16	8	0.65
*BMI*	33.2 ± 4.9	33.4 ± 5.7	33.2 ± 5.7	0.99
*Transverse Hernia Width (n)* *	5.8 ± 4.3 (15)	5.5 ± 3.5 (27)	4.7 ± 3.9 (15)	0.72
*Hernia Recurrence (n, yes)*	7	9	3	0.54

BMI, body mass index; TA, transverse; F, female

* number available at analysis timepoint

**Table 2.** Functional performance and quality of life scores across minimal, low, and moderate to high kinesiophobia groups. Results are median (interquartile range, IQR).

	Minimal (n=22)	Low (n=37)	Moderate to High (n=18)	P-value

*QUeST*	−2.0 (3.0)	−4.0 (7.0)	−5.0 (7.0)	0.17
*5xSTS (s)*	10.6 (3.6)	13.9 (3.2)	15.4 (8.50)	0.006
*HerQLes*	62.5 (40.4)	53.3 (31.7)	30.0 (33.3)	0.001

QUeST, quiet unstable sitting test; 5xSTS, five-time sit-to-stand; HerQLes, hernia related quality of life

## Data Availability

Data are available upon reasonable request.
